# Luoyutong Treatment Promotes Functional Recovery and Neuronal Plasticity after Cerebral Ischemia-Reperfusion Injury in Rats

**DOI:** 10.1155/2015/369021

**Published:** 2015-12-01

**Authors:** Ning-qun Wang, Li-ye Wang, Hai-ping Zhao, Ping Liu, Rong-liang Wang, Jue-xian Song, Li Gao, Xun-ming Ji, Yu-min Luo

**Affiliations:** ^1^Cerebrovascular Diseases Research Institute, Xuanwu Hospital, Capital Medical University, Ministry of Education, 45 Changchun Street, Beijing 100053, China; ^2^Department of Traditional Chinese Medicine, Xuanwu Hospital, Capital Medical University, Ministry of Education, 45 Changchun Street, Beijing 100053, China; ^3^Dongfang Hospital, Beijing University of Chinese Medicine, Beijing 100078, China; ^4^Key Laboratory of Neurodegenerative Diseases (Capital Medical University), Ministry of Education, 45 Changchun Street, Beijing 100053, China; ^5^Department of Neurology, Xuanwu Hospital, Capital Medical University, Ministry of Education, 45 Changchun Street, Beijing 100053, China

## Abstract

Luoyutong (LYT) capsule has been used to treat cerebrovascular diseases clinically in China and is now patented and approved by the State Food and Drug Administration. In this retrospective validation study we investigated the ability of LYT to protect against cerebral ischemia-reperfusion injury in rats. Cerebral ischemia-reperfusion injury was induced by middle cerebral artery occlusion followed by reperfusion. Capsule containing LYT (high dose and medium dose) as treatment group and Citicoline Sodium as positive control treatment group were administered daily to rats 30 min after reperfusion. Treatment was continued for either 3 days or 14 days. A saline solution was administered to control animals. Behavior tests were performed after 3 and 14 days of treatment. Our findings revealed that LYT treatment improved the neurological outcome, decreased cerebral infarction volume, and reduced apoptosis. Additionally, LYT improved neural plasticity, as the expression of synaptophysin, microtubule associated protein, and myelin basic protein was upregulated by LYT treatment, while neurofilament 200 expression was reduced. Moreover, levels of brain derived neurotrophic factor and basic fibroblast growth factor were increased. Our results suggest that LYT treatment may protect against ischemic injury and improve neural plasticity.

## 1. Introduction

Stroke is one of the leading causes of death worldwide and causes long-term disability. It has a significant impact upon health, well-being, and social interactions. Despite this, the available treatments remain limited and unsatisfactory and there is a considerable demand for novel therapies [1–3]. The use of Traditional Chinese Medicines (TCMs) in treating cerebral ischemia injury has increased in recent years and the regulatory pathways targeted by these medicines have been investigated [4–6]. A special group of medicinal plants and animals used in TCMs have been widely administered as patented Chinese medications to manage the symptoms of stroke, such as spasticity, altered muscle tone, and motor neuron excitability [7–10].

Neuronal plasticity involves changes in intracerebral structure and function in both gray matter and white matter. Gray matter consists mainly of neuronal cell bodies and unmyelinated axons. Within the gray matter, synaptic distribution and density can be measured by expression of the synaptic vesicle protein synaptophysin, while dendrites can be visualized using microtubule associated protein (MAP-2) as a marker. White matter is composed mainly of myelinated axons and myelin-producing oligodendrocytes as well as other glial cells. Myelin basic protein (MBP) is a marker of myelin and neurofilament 200 (NF200) is expressed in myelinated axons. The effect of stroke on gray matter has been well studied in the past, but more attention has been paid recently to white matter injury following stroke [11].

Luoyutong (LYT) capsule contains eight active TCM ingredients (Table 1) and is patented and approved by the State Food and Drug Administration in China. It has been used clinically for the treatment of acute and chronic cerebrovascular diseases. Previous studies showed it has a therapeutic effect of stroke [12, 13]. Our preliminary results suggest LYT plays a protective role in cerebral ischemia-reperfusion injury of rats [14]; however, the mechanism underlying the therapeutic effects of LYT in cerebrovascular diseases remains undefined. To address this, in this retrospective validation study, we investigated the protective effect and underlying mechanisms of LYT treatment in a rat model of cerebral ischemia-reperfusion injury.

## 2. Methods

### 2.1. Drug and Preparation

LYT (505 Pharmaceutical Co. Ltd., Lot number: 960815, Xianyang, Shanxi, China) was in the form of a dried superfine powder (≤10 *μ*m) composed of eight ingredients (Table 1), which were ground using a micronizer. LYT powder was prepared as a capsule, which was authenticated and standardized based on marker compounds in the Chinese Pharmacopoeia (Committee, 2005). The ingredients of the LYT capsule were carefully analyzed and quality-controlled. One gram of capsule is equivalent to 2.68 g of crude drug. The powder was dissolved in saline (0.08 g/mL) and stored at 4°C until subsequent use. Citicoline Sodium (CS) capsule (QILU Pharmaceutical Group Co. Ltd., Lot number: H20020220, Jinan, Shandong, China) was used clinically for cerebral ischemia, cerebral hemorrhage, and dementia because of its repair effect in recovery phase. Citicoline Sodium (CS) capsules were diluted in saline to a final concentration of 2 mg/mL as a positive control treatment group.

### 2.2. Animals

Male Sprague-Dawley rats weighing 280–300 g were purchased from Vital River Laboratory Animal Technology Co. Ltd. (Beijing, China). Animals were housed in an environmentally controlled room at 22 ± 2°C, with a 12 h/12 h light/dark cycle and were allowed free access to food and water throughout the entire study. The study was approved by the Institutional Animal Care and Use Committee of Capital Medical University and was in accordance with the principles outlined in the National Institutes of Health Guide for the Care and Use of Laboratory Animals.

### 2.3. Rat Model of Cerebral Ischemia-Reperfusion

Focal cerebral ischemia was induced in rats as previously described [15]. Briefly, rats were anesthetized with enflurane, and the right common carotid artery, the external carotid artery (ECA), and the internal carotid artery (ICA) were exposed. A 4-0 suture (diameter, 0.26 mm) with a blunted tip coated with poly-L-lysine was gently advanced into the ICA through the ECA. The suture was advanced 18–20 mm (reaching the origin of the right middle cerebral artery) beyond the carotid artery bifurcation. To allow reperfusion, the suture was slowly withdrawn after 1.5 h of middle cerebral artery occlusion (MCAO). Regional cerebral blood flow (0.5 mm anterior and 5.0 mm lateral to bregma) was monitored using laser Doppler flowmetry (PeriFlux System 5000, Perimed, Stockholm, Sweden) to ensure the occurrence of ischemia by MCAO. Rectal temperature was maintained at 37.0°C during and after surgery with a temperature-controlled heating pad (CMA 150 Carnegie Medicine, Sweden). Operations in the sham group were performed using the same surgical procedures, excepting the occlusion of the carotid arteries. All animals were housed in an air-conditioned room at 22 ± 2°C after recovering from anesthesia.

### 2.4. Grouping and Treatment

**Figure 1:**
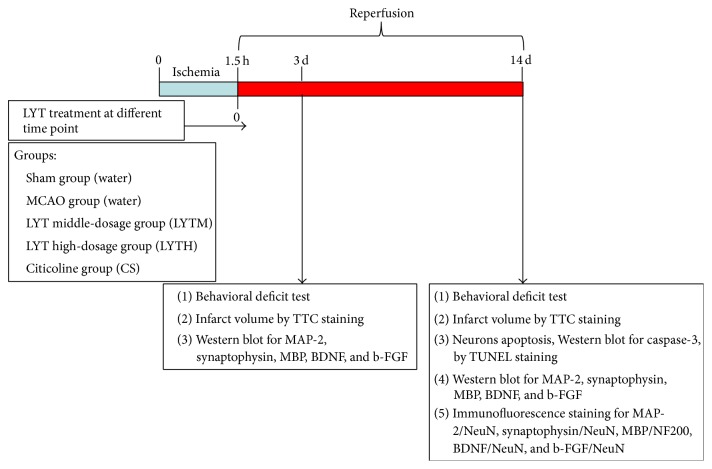
Schematic representation of the experimental procedures. (Luoyutong: LYT; middle cerebral artery occlusion: MCAO; 2,3,5-triphenyltetrazolium chloride: TTC; microtubule associated protein: MAP-2; myelin basic protein: MBP; brain derived neurotrophic factor: BDNF; basic fibroblast growth factor: b-FGF; neuron-specific nuclear protein: NeuN; neurofilament 200: NF200).

In total, 90 rats were randomly divided into five groups: sham group, MCAO group, medium dose (0.4 g/kg) of LYT (LYTM), high dose (0.8 g/kg) of LYT (LYTH), and CS (0.1 g/kg). Each group was divided into two subgroups, one treated for 3 days and the other for 14 days. Each subgroup contained nine animals; six were used for 2,3,5-triphenyltetrazolium chloride (TTC) staining to evaluate brain infarct and swelling volume and three rats were allocated for histological and Western blot analyses. A drug solution (2 mL) was administered by gavage and was calculated according to the body surface area based on the daily clinical dosage recommended for humans. Treatment started 30 min after reperfusion and was administered once a day for 3 or 14 days. Animals in the sham and MCAO groups received 2 mL 0.9% NaCl in the same manner. The experimental design is illustrated in Figure 1.

### 2.5. Evaluation of Motor Performance

Behavioral tests were performed blindly by a trained investigator to eliminate bias. Three tests were carried out to evaluate various aspects of neurological function. (1) The first one is Longa's score test [15], where a normal score is 0 and the maximum score is 5. (2) The second one is the 0–12 neurological score test, which included the postural reflex and forelimb placing test, graded on a scale of 0 to 12. The postural reflex test examined the upper body posture while the animal was suspended by the tail and the forelimb placing test evaluated the response of the forelimb to visual, tactile, and proprioceptive stimuli. (3) The third one is the modified foot fault test [16, 17] which recorded the number of times the forelimb was misplaced, causing the rat to fall through the grid.

### 2.6. Measurement of Infarct Volume

The animals were sacrificed 3 or 14 days after reperfusion in a carbon dioxide chamber and the brains were quickly removed and sectioned into six consecutive coronal slices of 2 mm thickness. The slices were stained by immersing in 2% TTC for 30 min at 37°C, followed by fixation in 8% formalin. The border between infarcted and noninfarcted tissues was outlined with an image analysis system. The area of infarction was measured by subtracting the area of the nonlesioned ipsilateral hemisphere from that of the contralateral hemisphere based on Swanson's method [18]. The infarct volume was calculated as follows: 100%  ×  (contralateral hemisphere volume − nonlesioned ipsilateral hemisphere volume)/contralateral hemisphere volume. The swelling volume was calculated as follows: 100%  ×  (ipsilateral hemisphere volume − contralateral hemisphere volume)/contralateral hemisphere volume.

### 2.7. Terminal Deoxynucleotidyl Transferase dUTP Nick End Labeling (TUNEL)

Rats were euthanized 14 days after reperfusion with intraperitoneal injections of chloral hydrate (300 mg/kg) and were perfused transcardially with 4% w/v paraformaldehyde in phosphate-buffered saline (PBS). The brains were dehydrated in 30% sucrose in 4% formaldehyde in PBS. Frozen brains were sectioned coronally. Apoptotic cell death was detected using the In Situ Cell Death Detection Kit, POD (Roche, San Francisco, CA, USA), according to the manufacturer's instructions.

### 2.8. Western Blotting Analysis

The forward fontanelle, cut at a coronal slice thickness of 2 mm from the optic chiasma, was harvested 3 or 14 days after reperfusion. Samples were homogenized in lysis buffer (50 mM Tris-HCl, pH 7.5, 100 mM NaCl, and 1% Triton X-100) containing protease inhibitors (aprotinin, leupeptin, phenylmethylsulfonyl fluoride, and pepstatin) and phosphatase inhibitors (Sigma cocktail, Sigma-Aldrich, St. Louis, MO, USA). For each sample, 100 *μ*g total protein was resolved by sodium dodecyl sulfate polyacrylamide gel electrophoresis, followed by electrophoretic transfer to polyvinylidene difluoride membranes. Membranes were incubated overnight at 4°C in a 1 : 1000 dilution of primary antibodies against caspase-3 (Abcam, Cambridge, UK), synaptophysin (Abcam), MAP-2 (Cell Signaling Technology, Boston, MA, USA), MBP (Abcam), brain derived neurotrophic factor (BDNF) (Abcam), or basic fibroblast growth factor (b-FGF) (Abcam). Chemiluminescent detection of antigens was performed following incubation with horseradish peroxidase-conjugated secondary antibodies (Santa Cruz Biotechnology, Santa Cruz, CA, USA) for 60 min at room temperature using an enhanced luminescence kit (Millipore, Billerica, MA, USA).

### 2.9. Immunofluorescence

Rats were euthanized 14 days after reperfusion with intraperitoneal injections of chloral hydrate (300 mg/kg) and perfusion with cold saline. The brains were dehydrated in 30% sucrose and 4% formaldehyde in PBS. Frozen brains were sectioned coronally for immunohistochemistry. Following incubation for 2 h in a blocking solution containing 1% bovine serum albumin, 2% normal goat serum, 0.3% Triton X-100, and 5% nonfat dry milk in PBS, sections were labelled using primary antibodies against synaptophysin (Abcam), MAP-2 (Cell Signaling Technology), MBP (Abcam), NF200 (Abcam), BDNF (Abcam), b-FGF (Abcam), or neuron-specific nuclear protein (NeuN) (Millipore) at a dilution of 1 : 50. Sections were then incubated in fluorescently conjugated secondary antibodies (Alexa 488/Alexa 594-conjugated anti-mouse/anti-rabbit IgG) and fluorescence was detected using a fluorescence microscope (Carl Zeiss, Jena, Germany).

### 2.10. Statistical Analysis

Statistical analysis was performed using SPSS 11.0 (SPSS, Chicago, IL, USA). Data were expressed as means ± SEM and were statistically analyzed by one-way analysis of variance (ANOVA) followed by LSD post hoc test. *P* values less than 0.05 were considered statistically significant.

## 3. Results

### 3.1. LYT Decreases Infarct Volume and Improves Neurological Function following Cerebral Ischemia-Reperfusion Injury in Rats

**Figure 2:**
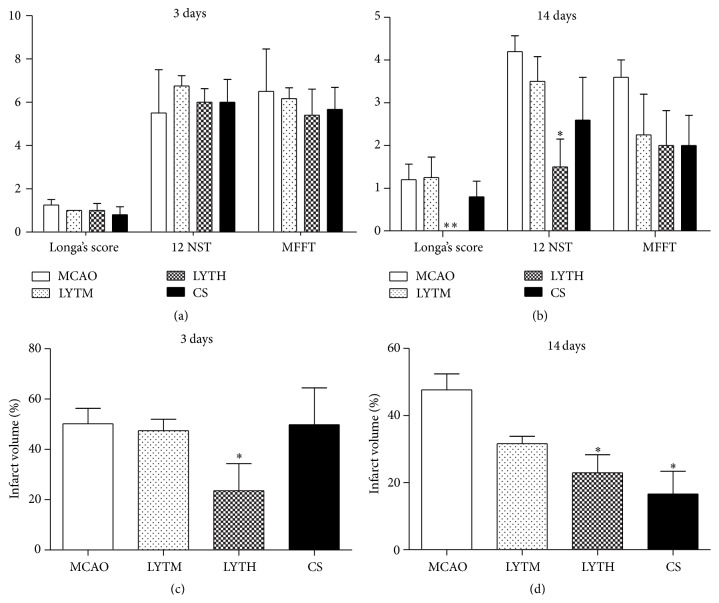
LYT (Luoyutong) decreases infarct volume and improves neurological function in rats following ischemic reperfusion. (a) Longa's score, 0–12 neurological score test, and modified foot fault test of MCAO (middle cerebral artery occlusion), LYTM (medium dose (0.4 g/kg) of LYT), LYTH (high dose (0.8 g/kg) of LYT), and CS (Citicoline Sodium) 3 days after MCAO. (b) Longa's score, 0–12 neurological score test, and modified foot fault test of MCAO, LYTM, LYTH, and CS 14 days after MCAO. (c) Infarct volume (%) of MCAO, LYTM, LYTH, and CS 3 days after MCAO. (d) Infarct volume (%) of MCAO, LYTM, LYTH, and CS 14 days after MCAO; ^*∗*^ *P* < 0.05 versus* MCAO* and ^*∗∗*^ *P* < 0.01 versus* MCAO*.

Behavioral tests and measurements of the infarct volume were used to evaluate the neurological outcome. The scores in all behavioral tests performed 3 days after reperfusion were not significantly different among groups (Figure 2(a)). There was a significant reduction in Longa's score test and the 0–12 neurological score test performance on day 14 in the LYTH group compared to the MCAO group (Longa's, *P* < 0.01; 0–12 neurological score test, *P* < 0.05), but the effect was not obvious in LYTM group and CS group, indicating improved neurological function in the LYTH group. Scores in the modified foot fault test were lower following all treatments (Figure 2(b)).

Cerebral infarct volume was measured by TTC staining (Figures 2(c) and 2(d)). The infarct volume in the LYTH group was significantly reduced compared to the MCAO group 3 and 14 days after ischemia-reperfusion injury (Figures 2(c) and 2(d), *P* < 0.05). LYTM did not impact infarct volume as significantly as LYTH. CS treatment only decreased infarct volume after 14 days of treatment (Figure 2(d), *P* < 0.05).

### 3.2. LYT Reduces Apoptosis in Neurons and Decreases the Level of Caspase-3

**Figure 3:**
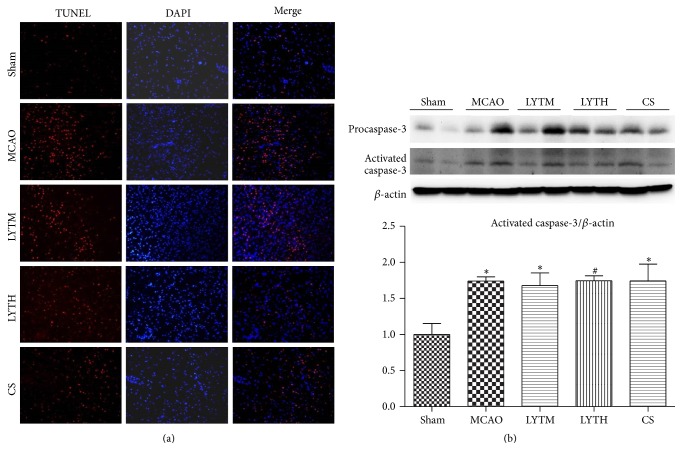
LYT (Luoyutong) reduces neuronal apoptosis and decreases the level of caspase-3. (a) Neuronal apoptosis in the ipsilateral cortex was detected by terminal deoxynucleotidyl transferase dUTP nick end labeling and 4′ 6-diamidino-2-phenylindole double staining 14 days after ischemic reperfusion. (b) Expression of activated caspase-3 was detected by Western blot 14 days after ischemic reperfusion. *n* = 3. ^*∗*^ *P* < 0.05 versus* Sham* and ^#^ *P* < 0.05 versus* MCAO*.

To measure apoptosis, TUNEL staining was performed in the cortex of ipsilateral cerebral tissue 14 days after reperfusion (Figure 3(a)). The number of apoptotic neurons in the MCAO group was significantly higher than that in the sham group. LYTH, LYTM, and CS treatment significantly decreased the number of TUNEL-positive cells compared to the MCAO group (Figure 3(a)). We also quantified the expression of activated caspase-3 in cerebral tissue 14 days after reperfusion (Figure 3(b)). Activated caspase-3 expression in the MCAO group was significantly higher than that in the sham group (Figure 3(b), *P* < 0.05). The expression of activated caspase-3 was reduced by LYTH, LTYM, and CS treatment, but a significant reduction was only observed in the LYTH group (Figure 3(b), *P* < 0.05).

### 3.3. LYT Protects against Ischemia-Reperfusion Injury by Enhancing Neural Plasticity

To investigate the effect of LYT on neural plasticity following ischemia-reperfusion injury in rats, we measured the levels of synaptophysin, MAP-2, and MBP by Western blotting. We also analyzed the colocalization of synaptophysin and MAP-2 with NeuN in the cortex and MBP with NF 200 in the corpus callosum by immunofluorescence.

**Figure 4:**
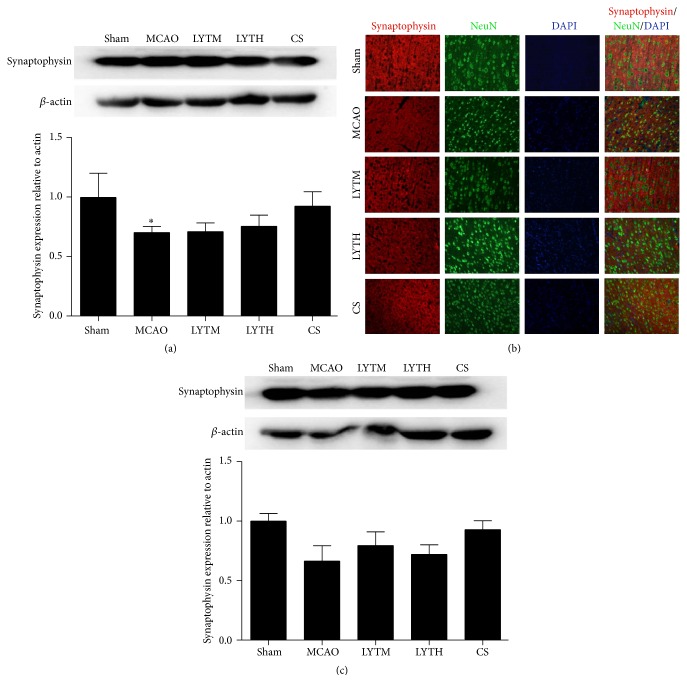
LYT (Luoyutong) induced upregulation of synaptophysin expression after MCAO (middle cerebral artery occlusion). Western blot detection and quantitative analysis of (a) synaptophysin expression 3 days after MCAO and (c) synaptophysin expression 14 days after MCAO. (b) Representative immunofluorescence images showing colocalization of synaptophysin (red) and NeuN (neuron-specific nuclear protein) (green) in the cortex. Blue DAPI staining indicates the nuclei. *n* = 3. ^*∗*^ *P* < 0.05 versus* Sham*.

Synaptophysin expression was decreased in the MCAO group compared with the sham group 3 days after reperfusion (Figure 4(a), *P* < 0.05). Both of LYT and CS had no obvious effect on it (Figure 4(a), *P* < 0.05). After 14 days, there were no significant differences between sham, MCAO, and treatment groups (Figure 4(c)). Immunofluorescence revealed that synaptophysin staining appeared as punctate-like or bouton-like distribution pattern with high immunofluorescence signal; staining of NeuN was shiny and sparkly. MCAO disrupted the localization of synaptophysin and dampened brightness of NeuN in the cortex, and this effect was reversed after 14 days of LYT and CS treatment (Figure 4(b)).

**Figure 5:**
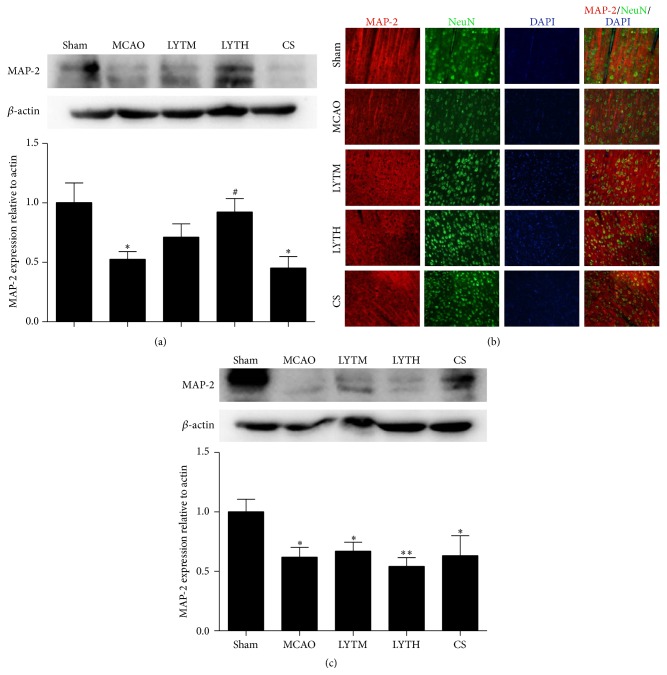
LYT (Luoyutong) induced upregulation of MAP-2 (microtubule associated protein) expression after MCAO (middle cerebral artery occlusion). Western blot detection and quantitative analysis of (a) MAP-2 expression 3 days after MCAO and (c) MAP-2 expression 14 days after MCAO. (b) Representative immunofluorescence images showing colocalization of MAP-2 (red) and NeuN (neuron-specific nuclear protein) (green) in the cortex. Blue DAPI staining indicates the nuclei. *n* = 3. ^*∗*^ *P* < 0.05 versus* Sham*, ^*∗∗*^ *P* < 0.01 versus* Sham*, and ^#^ *P* < 0.05 versus* MCAO*.

Western blot analysis showed a reduced expression of MAP-2 in the MCAO group compared with the sham group 3 and 14 days after reperfusion (Figures 5(a) and 5(c), *P* < 0.05). MAP-2 levels were increased by 3 days of LYTH treatment after reperfusion (Figure 5(a), *P* < 0.05), whereas administration of LYTM and CA did not rescue MAP-2 expression. After 14 days, there was no difference between MCAO and treatment groups (Figure 5(c)). As shown in immunofluorescence, the expression of MAP-2 in sham group was shown in dendrites; it became irregular, discontinuous, and weaker in MCAO and it was improved by LYTH and LYTM treatment but not CS treatment (Figure 5(b)).

**Figure 6:**
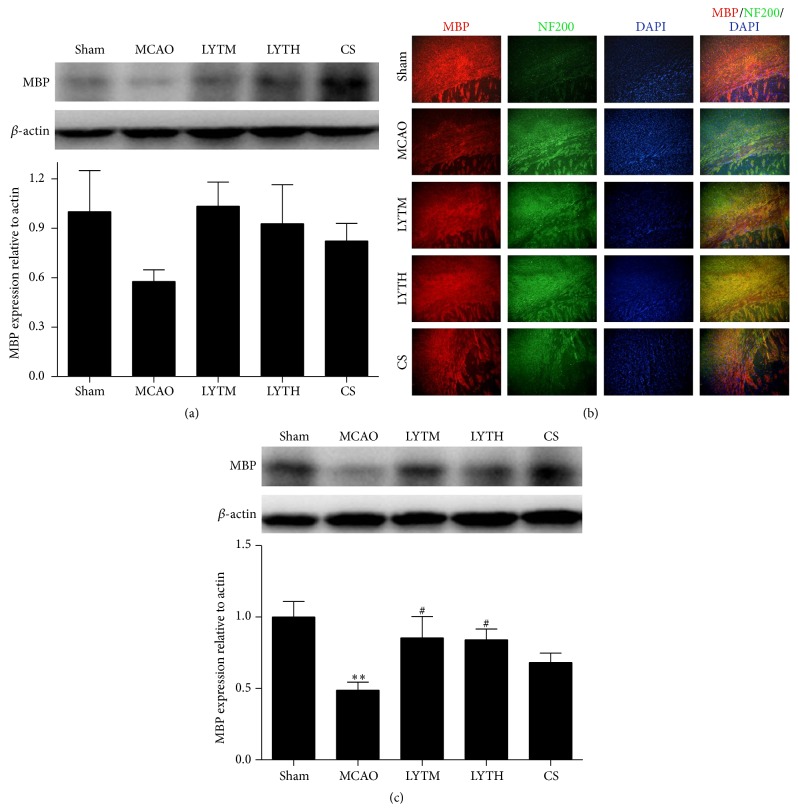
LYT (Luoyutong) induced upregulation of MBP (myelin basic protein) expression after MCAO (middle cerebral artery occlusion). Western blot detection and quantitative analysis of (a) MBP expression 3 days after MCAO and (c) MBP expression 14 days after MCAO. (b) Representative immunofluorescence images showing colocalization of MBP (red) and NF200 (neurofilament 200) (green) in the cortex. Blue DAPI staining indicates the nuclei. *n* = 3. ^*∗∗*^ *P* < 0.01 versus* Sham* and ^#^ *P* < 0.05 versus* MCAO*.

The expression of MBP was lower in the MCAO group compared to the sham group 3 and 14 days after reperfusion, but this was only statistically significant after 14 days (Figures 6(a) and 6(c), *P* < 0.01). We observed an increased MBP expression after 3 and 14 days of treatment with LYT and CS (Figures 6(a) and 6(c)); similarly the significant difference was only significant after 14 days of LYT treatment (Figure 6(c), *P* < 0.05). MBP immunoreactivity was affected mostly by myelin sheaths in the sham group. However, low immunoreactivity was detected for MBP after MCAO, while NF200 staining increased in intensity in the MCAO group. These effects were reversed by all treatments (Figure 6(b)).

Taken together, these findings demonstrated that the different treatments protected neural plasticity following ischemia-reperfusion injury, particularly the high dose of LYT.

### 3.4. LYT Treatment Upregulated BDNF and b-FGF after Ischemia-Reperfusion Injury

**Figure 7:**
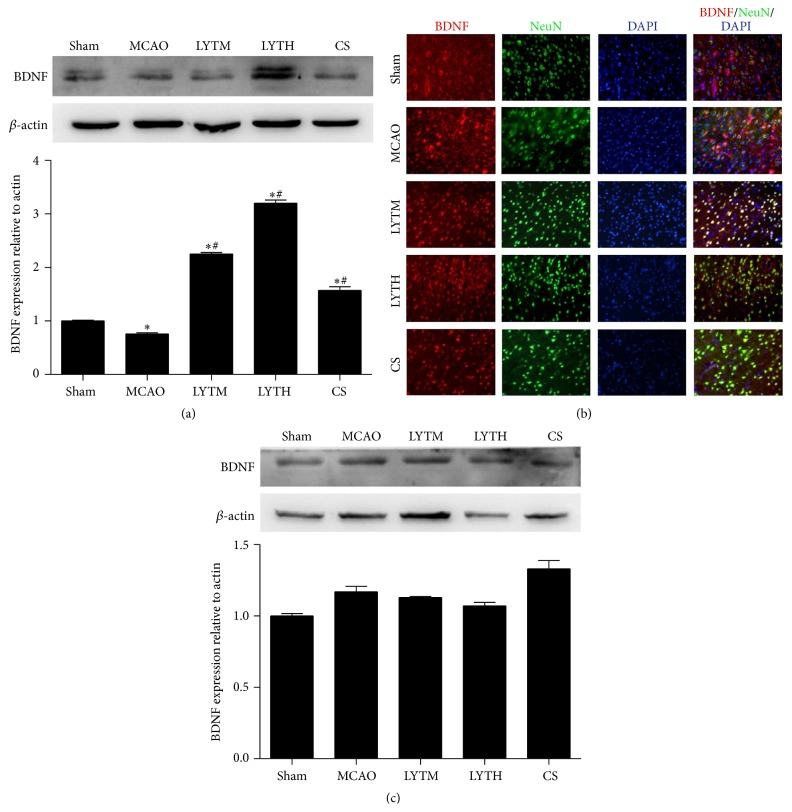
LYT (Luoyutong) induced upregulation of BDNF (brain derived neurotrophic factor) expression after MCAO (middle cerebral artery occlusion). Western blot detection and quantitative analysis of (a) BDNF expression 3 days after MCAO and (c) BDNF expression 14 days after MCAO. (b) Representative immunofluorescence images showing colocalization of BDNF (red) and NeuN (neuron-specific nuclear protein) (green) in the cortex. Blue DAPI staining indicates the nuclei. *n* = 3. ^*∗∗*^ *P* < 0.01 versus* Sham* and ^#^ *P* < 0.05 versus* MCAO*.

**Figure 8:**
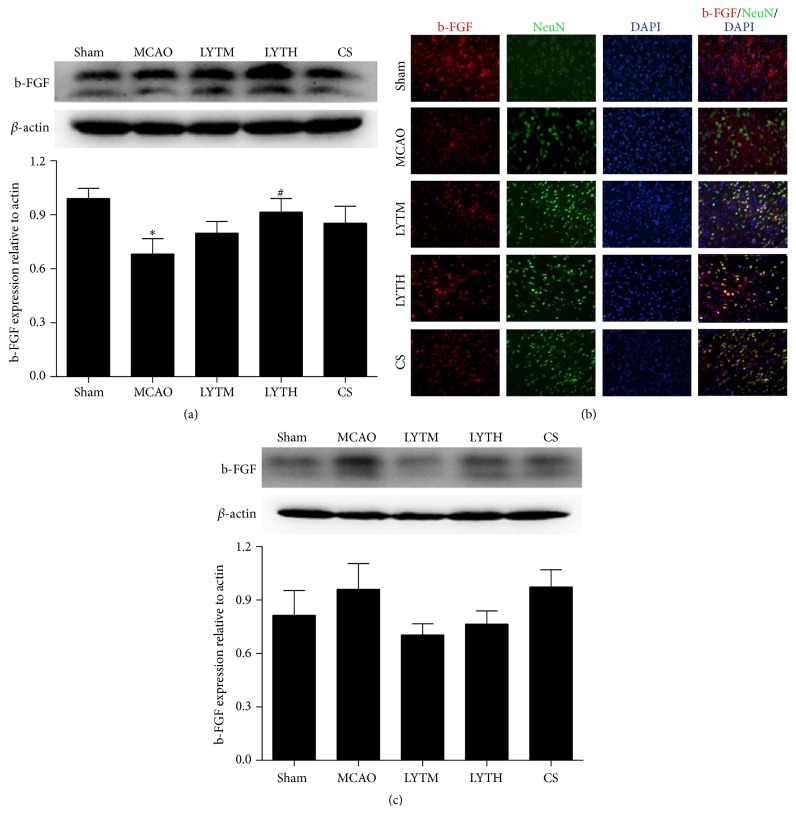
LYT (Luoyutong) induced upregulation of b-FGF (basic fibroblast growth factor) expression after MCAO (middle cerebral artery occlusion). Western blot detection and quantitative analysis of (a) b-FGF expression 3 days after MCAO and (c) b-FGF expression 14 days after MCAO. (b) Representative immunofluorescence images showing colocalization of b-FGF (red) and NeuN (neuron-specific nuclear protein) (green) in the cortex. Blue DAPI staining indicates the nuclei. *n* = 3. ^*∗∗*^ *P* < 0.01 versus* Sham* and ^#^ *P* < 0.05 versus* MCAO*.

To investigate the effect of LYT treatment on the expression of growth factors, we measured BDNF and b-FGF expressions by Western blot 3 and 14 days after reperfusion. BDNF expression was significantly lower in the MCAO group compared to the sham group 3 days after reperfusion (Figure 7(a), *P* < 0.05). LYT and CS treatment dramatically increased BDNF expression 3 days after reperfusion and it was even higher than that in the sham group (Figure 7(a), *P* < 0.05). After 14 days, no significant differences were observed in BDNF expression between the groups (Figure 7(c)). BDNF positive cells partly colocalized with NeuN as demonstrated by immunofluorescence; the staining of BDNF was not different among groups (Figure 7(b)).

b-FGF expression was also lower in the MCAO group compared to the sham group 3 days after reperfusion (Figure 8(a), *P* < 0.05) and only treatment with LYTH could reverse this effect. No difference in b-FGF expression was observed 14 days after reperfusion (Figure 8(c)). Immunofluorescence revealed that a portion of b-FGF showed colocalization with NeuN in the cortex; MCAO and treatment had no effect on its expression (Figure 8(b)).

## 4. Discussion

Clinical research showed that LYT has a therapeutic effect of stroke [12, 13]; however, the mechanism underlying the therapeutic effects remains undefined. To address this, LYT was applied to rat model of cerebral ischemia-reperfusion injury. As expected, LYTH treatment could improve neurologic function and reduce infarction volume, which is more significant than CS treatment group. Further studies revealed that the protective effect of LYT is potentially related to the improvement of neural plasticity and upregulation of BDNF and b-FGF. The measurement of infarction volume showed that LYT reduces infarct volume significantly in both acute stage (3 d) and restoration stage (14 d). It is noteworthy that CS has no effect on it in acute stage (3 d). Although CS is widely used in clinic, its treatment still has limitations. Moreover, LYT showed the improvement effect of neurological function at 14 days after ischemia-reperfusion injury in accord with clinical research [12, 13]. We speculate that early inhibition of infarction volume is more advantageous to the late reply of neurological function.

In order to make clear whether the therapeutic effect of LYT depends on antiapoptosis function, we measured apoptosis by TUNEL and quantified the expression of activated caspase-3 in cerebral tissue 14 days after reperfusion. Both CS and LYTH have pronounced influence on apoptosis, but only LYTH could reduce the expression of activated caspase-3. Since caspase-3 is not only a known regulator of apoptosis, it can also influence neural plasticity [19]. Therefore, the downregulation of caspase-3 in response to LYT treatment not only may influence apoptosis exclusively but also can promote neural plasticity.

Neural plasticity is important for neurological function recovery after reperfusion, and neural plasticity has different performance at different time points. Neural plasticity plays a vital role in ischemic injury [20], depression, and memory [21, 22]. MAP-2 is dendritic marker and synaptophysin is synaptic marker. Synaptophysin regulates activity-dependent synapse formation [23]. MAP-2 nucleates and stabilizes microtubules, regulates organelle transport, and anchors regulatory proteins within neurons to regulate process outgrowth, synaptic plasticity, and apoptosis [24]. In present study, our results demonstrated that synaptophysin and MAP-2 levels decrease after MCAO, in agreement with previous findings [25, 26]. Meanwhile MCAO disorganized the arrangement structure of MAP-2, but the arrangement structure is the foundation of its function. The result showed that only LYTH could increase the level of MAP-2, and this function only embodied in 3 days. CS and LYTM do not have obvious effect on it. But all of the treatments have no effect on synaptophysin. These findings suggest that LYTH can enhance neural plasticity through regulating the expression of MAP-2 following ischemia-reperfusion injury in gray matter. Cerebral white matter is also highly vulnerable to ischemia, and white matter is subject to a novel form of neural plasticity which is termed “myelin plasticity” [27]. Changes in MBP and NF200 expression can be indicative of white matter injury following MCAO [28]. The present study is consistent with previous reports on MBP and NF200 expression changes after ischemia-reperfusion injury. After 14 days, the change is more prominent. LYT treatment can reverse these effects and the improvement of LYT is more remarkable after 14 days. However, the effect of CS is not obvious. Our results revealed a regulatory role of LYT in white matter plasticity following cerebral ischemia-reperfusion injury, while CS treatment could not improve white matter injury.

In addition, LYT treatment upregulates the expression of BDNF and b-FGF following ischemia-reperfusion injury, both of which are critical mediators of neural plasticity and survival [19, 29–35]. The influence of MCAO and the intervention effect of treatment only reflect on the third day. The results from the expression of BNDF and b-FGF indicate that LYT is stronger than CS in regulating growth factors, especially LYTH.

In summary, we have provided evidence that LYT has a protective effect after ischemia-reperfusion injury in rats by suppressing neuronal apoptosis and repairing neural plasticity of both gray matter and white matter. These effects may be mediated by regulating caspase-3, BDNF, and b-FGF expressions. Further elucidation of the signaling pathways involved is required. It is likely that the herbal ingredients of LYT act synergistically, but the relative contribution of each herb to the beneficial outcome has not yet been defined.

## Figures and Tables

**Table 1:** LYT ingredients.

Ingredients (Latin name)	Family	Part used	Processing	Amount used %
Plants				
*Astragalus membranaceus* (Fisch.) Bge.	Leguminosae	Dried root	Extraction	18.159
*Ligusticum chuanxiong* Hort.	Umbelliferae	Root and rhizome	Extraction	9.079
*Spatholobus suberectus* Dunn	Papilionaceae	Rattan and stem	Extraction	13.681
Insects				
*Pheretima vulgaris* Chen	Guaibasauridae	Dried body	Farina	27.238
*Whitmania pigra* Whitman	Hirundinidae	Dried body	Farina	13.681
*Buthus martensii* Karsch	Buthidae	Dried body	Farina	5.472
*Scolopendra subspinipes mutilans* L. Koch	Psittacidae	Dried body	Farina	4.477
*Bombyx mori* Linnaeus	Bombycidae	Dried body	Farina	8.208
